# 

**Published:** 1871-04

**Authors:** 


ORIGINAL CONTRIBUTIONS.

Atmospheric Germs and their Relation to Disease. By Frank H. Davis, M.D., 193
Observations on the Sanitary Condition with Remarks on the Pathology and Treatment of some of the Diseases of Southern Indiana. By Robert Robson, M.D., New Harmony, Ind., .......... 199
A Case of Cauliflower. By E. R. Willard, M.D., Wilmington, Illinois, .......... 205
Tea-Leaves as an Application to Burns and Scalds. By W. H. Searles, M.D., Warsaw, Wis., .......... 207

THE CLINIC.

Clinical Cases in Medical Wards of Mercy Hospital. By Prof. Davis. From Notes by S., .......... 208

CORRESPONDENCE.

Morphia as a Parturient. By George H. Vance, M.D., .......... 215

Hydrate of Chloral. H. N. Hurlbut, M.D., 216

PROCEEDINGS OF SOCIETIES.

Alumni Association of the Chicago Medical College, .......... 216
The Transactions of the Esculapian Society of the Wabash Valley, at its Annual Session held at Tuscola, Illinois, October 26-7, 1870, .......... 217

SELECTIONS.

On Acquired Syphilis. By William Wilson, M.D., M.Ch., Q.U.I., .......... 222

ABRIDGMENTS FROM EXCHANGES.

Fibrous Stricture of the Rectum relieved by Incisions and Elastic Pressure, .......... 226
Skimmed Milk as a Diet in Disease, .......... 229
Amputation at the Hip-Joint, .......... 231
Inflammation of the Mastoid Process, .......... 231

BOOK NOTICES.

The Change of Life in Health and Disease, .......... 232
A Treatise on the Chronic Inflammations and Displacements of the Unimpregnated Uterus, .......... 232
The Journal of the Gynæcological Society, 233

EDITORIAL.

Sutures into the Uturus after Cæsarian Section, .......... 233
Medical Legislation, .......... 236
Chicago Medical College Commencement 240
American Medical Association, .......... 242
Illinois State Medical Society .......... 243
Summer Course of Medical Instruction, .. 243
Alumni Association of the Medical Department of the University of the City of New York, .......... 243
Postponement, .......... 244
Removal, .......... 244
An operation for the Cure of Obstructive Dysmenorrhœa, .......... 244
Digitalis Leaves in Orchitis, .......... 244
Chronic Catarrh, .......... 244
To Disguise the Taste of Cod-Liver Oil, .. 244
Death from Chloroform, .......... 244
Mortality for the Month of February, 1871 245
Treatment of Delirium Tremens, .......... 247
Oxygen Gas, .......... 246
Cauterization in Diphtheria, .......... 249
Sympathetic Nervous Troubles from the Presence of a Tenia, .......... 249
Thoracentesis, .......... 250
Perforating Ulcer of the Bladder, .......... 251
Rules in Operating on the Tongue, .......... 251
Spontaneous Origin of Enteric Fever .... 252
Albuminuric Retinitis, .......... 253
Intracranial Abscess Cured by Trephining, 253
Chloroform in Labor, .......... 253
Gonorrhœa Produceable by Chancre, .... 254
Death from Inhalation of Ether, .......... 254
Small-Pox in London, .......... 254

